# The use of α- or β-blockers to ameliorate the chronic stress of captivity in the house sparrow (*Passer domesticus*)

**DOI:** 10.1093/conphys/cow049

**Published:** 2016-10-15

**Authors:** Clare Parker Fischer, L. Michael Romero

**Affiliations:** Tufts University, Department of Biology, 163 Packard Avenue, Medford, MA 02155, USA

**Keywords:** Avian, captivity, glucocorticoid, heart rate, stress

## Abstract

When wild animals are brought into captivity for the first time, they frequently develop chronic stress symptoms. Animals can develop glucocorticoid dysregulation or changes in the sympathetic nervous system over the course of the first week in captivity. By blocking the action of epinephrine and norepinephrine using α- or β-blockers, we hoped to reduce the degree of chronic stress symptoms exhibited by newly captured house sparrows. We measured corticosterone, heart rate and heart rate variability in 24 house sparrows (*Passer domesticus*) over the first week of captivity. The birds were treated with saline, propranolol (a β-blocker) or phentolamine (an α-blocker) for the first 3 days of captivity. We also compared newly captured animals with animals that had been held in captivity for 1 month. During the first week of captivity, baseline corticosterone increased, but that increase was blocked by propranolol. Heart rate was not different between the treatment groups, but it was higher during the first week than after 1 month in captivity. Sympathetic nervous system activity (as measured by heart rate variability) decreased over the first week of captivity, but was not affected by treatment. β-Blockers, but not α-blockers, might help to improve some symptoms of chronic stress in newly captured animals.

## Abstract (precis)

Wild animals can become chronically stressed when they are brought into captivity. Newly captured animals have altered glucocorticoid concentrations and increased heart rates, in part because of high concentrations of epinephrine and norepinephrine. β-Blockers (but not α-blockers) might help to reduce some chronic stress symptoms in newly captured wild house sparrows.

## Introduction

When wild animals are brought into captivity, they are faced with circumstances unlike any they have experienced before. Confinement, artificial light conditions, altered diet, the presence of and handling by humans, and other factors contribute to unpredictable and uncontrollable living conditions (sources of stress in captivity reviewed by Morgan and Tromborg, 2007). Not surprisingly, chronic stress develops in wild animals of many species when they are brought into captivity (e.g. Terio *et al*., 2004; Cabezas *et al*., 2007; Dickens *et al*., 2009; Adams *et al*., 2011; Lattin *et al*., 2012). Chronic stress occurs when stressors are ongoing or repeated, and the physiological systems that are normally important for surviving and recovering from negative events become dysregulated and begin to cause problems. Although the effects of captivity on glucocorticoid hormones are relatively well documented in birds (Wingfield *et al*., 1982; Dickens *et al*., 2009; Adams *et al*., 2011; Lattin *et al*., 2012), less is known about the catecholamine side of the response. When animals overproduce epinephrine (E) and norepinephrine (NE) during the first few days of captivity, this may cause the production of these hormones to become dysregulated. They may produce too much E or NE at rest (resulting in a heart rate that is too fast) or too little E or NE when it is needed. Poorly regulated E and NE production could potentially have negative health outcomes. By temporarily blocking the action of E and NE using receptor antagonists, we expected to reduce chronic stress symptoms in captive house sparrows. Not only will this study help to uncover the role of E and NE in development of chronic stress, but also it may provide useful tools for reducing chronic stress in birds brought into captivity for conservation or research.

There are two major hormonal systems in play during the stress response: the release of glucocorticoids [in birds, corticosterone (CORT)] and the release of catecholamine hormones (E and NE), resulting in an increase of sympathetic nervous system (SNS) activation. In an acute stress response, CORT binds to receptors throughout the body, resulting in a shift in energy use away from reproduction, growth and other long-term investments and towards immediate survival and recovery (reviewed by Sapolsky *et al*., 2000). The release of E and NE from the adrenal medulla results in a very rapid increase in heart rate (the startle response), as well as changes in the respiratory, vascular and digestive systems. The two systems are independently regulated in birds (Nephew and Romero, 2003; Dickens *et al*., 2006) but they do interact. Corticosterone increases catecholamine secretion in birds (Zachariasen and Newcomer, 1974; Mahata and Ghosh, 1991) and increases responsiveness to catecholamines (reviewed by Sapolsky *et al*., 2000). Epinephrine and NE stimulate the release of CORT by triggering the release of adrenocorticotrophic hormone (ACTH) from the pituitary (mammals: Bugajski *et al*., 1995; Mezey *et al*., 1983; birds: Rees *et al*., 1985).

The role of CORT in acute and chronic stress has been extensively studied in wild animals in both field and laboratory conditions. Chronic stress typically results in (and is often defined by) changes in CORT regulation following repeated exposures to a stressor. In many bird species, captivity has been found to cause changes in glucocorticoid concentrations, although the direction of change is species specific (Dickens *et al*., 2009; Adams *et al*., 2011; Lattin *et al*., 2012). However, the relationship between catecholamine hormones and chronic stress has been relatively understudied in wild birds. Dysregulation of the SNS may occur following high E/NE signalling during the first few days of captivity. Dickens and Romero (2009) documented elevated heart rate, elevated SNS activity and a drastically reduced startle response in European starlings during the first days of captivity. A potentially diminished startle response during the transition to captivity has been reported in other animals. For example, newly captured bighorn sheep had lower plasma concentrations of E and NE during an acute stressor than sheep raised in captivity (Coburn *et al*., 2010). Captive harbor porpoises also had lower plasma E and NE after being netted and sampled than free-living porpoises (Siebert *et al*., 2011). However, we do not know whether an impaired startle response is a general response to captivity amongst many species.

In this study, we developed a new harness-mounted system for recording heart rate in house sparrows, a much smaller (~27 g) species than the European starling. We then chemically blocked catecholamine receptors during the first few days of captivity to assess the role of E and NE in developing chronic stress symptoms. There are two major types of E/NE receptors, α- and β-receptors, which are present in a variety of tissues, including the central nervous system. α-receptors are present in the vascular system and are responsible for constriction of smooth muscle, such as that found in peripheral blood vessels (Ahlquist, 1948; Minneman *et al*., 1981). β-receptors are present on the heart and smooth muscle. They cause an increase in heart rate, as well as relaxation of smooth muscle, such as that found in bronchi (Ahlquist, 1948; Minneman *et al*., 1981). We tested the effects of blocking either α-receptors (using 3 mg/kg phentolamine injected intramuscularly in the pectoralis) or β-receptors (using 3 mg/kg intramuscular propranolol) in house sparrows during the first week of captivity.

We expected that the chronic stress of captivity would cause the following changes in the physiology of newly captured house sparrows. First, Lattin and colleagues (2012) previously documented the effects of captivity on mass and glucocorticoid concentrations in house sparrows. We expected to replicate their results and see weight loss, increased baseline CORT and increased CORT negative feedback after 1 week in captivity. Second, Dickens and Romero (2009) documented changes in heart rate over the first week of captivity in European starlings. We expected that house sparrows likewise would show high heart rate that would decrease over the first week. Third, a high heart rate can be the result of increased SNS activity (and therefore higher concentrations of E and NE). However, the parasympathetic nervous system (PNS) also regulates heart rate; a high heart rate could alternatively indicate less PNS activity. We can tease this apart by examining heart rate variability (HRV). The PNS causes the heart rate to vary with every breath cycle; variation caused by the SNS occurs on a longer time scale (Stauss, 2003). Therefore, by comparing the beat-to-beat intervals on a short time scale, we can determine how much influence there is of the PNS relative to the SNS (high HRV indicates more PNS and less SNS activity; Korte *et al*., 1999; Perini and Veicsteinas, 2003; Cyr *et al*., 2009). Dickens and Romero (2009) found that newly captured European starlings had low HRV, which increased over the course of the first week. We expected to see a similar pattern in house sparrows. Fourth and finally, Dickens and Romero (2009) documented a drastically reduced startle response in newly captured European starlings. We expected house sparrows likewise to have a suppressed startle response. Our predictions for the SNS were based on studies in European starlings, the only previous studies to our knowledge of heart rate in newly captive passerines. However, house sparrows and European starlings do not have identical corticosterone responses to chronic stress (Rich and Romero, 2005; Lattin *et al*., 2012). It was reasonable to expect that house sparrows would also show somewhat different heart rate changes in response to captivity.

In addition to replicating previous studies on chronic captivity stress, we designed our study to determine how E and NE impacted the development of chronic stress symptoms and whether these symptoms could be ameliorated by blocking the adrenergic receptors. We hypothesized that blocking the adrenergic receptors would speed up acclimation to captivity. When comparing propranol-treated, phentolamine-treated and saline-treated animals, we predicted that: (i) propranolol- and phentolamine-treated birds would show a more rapid decrease in resting heart rate over the course of the first week in captivity than saline-treated birds; (ii) propranolol- and phentolamine-treated birds would show a more rapid increase in HRV over the course of the first week than saline-treated birds; and (iii) propranolol- and phentolamine-treated birds would show a more robust startle response after 7 days compared with saline-treated birds. If blockage of E/NE reduces all chronic stress symptoms, not only those related to the cardiovascular system, we would expect treated birds to have a reduced baseline CORT compared with saline-treated birds, as well as potential differences in negative feedback in the CORT response and weight loss.

## Materials and methods

### Drug validations and heart rate after 1 month in captivity

Eight house sparrows were captured in Medford, MA, USA and held in captivity for 4 weeks. After this period of acclimation, the birds were fitted with heart rate transmitter harnesses (see subsection “Heart rate transmitter harnesses”). Resting heart rate and heart rate variability were recorded for 3 min every 2 h for 3 days while the birds were left undisturbed except for normal animal care. These birds were also used to test the acute effect of propranolol, phentolamine and saline on heart rate. Heart rate was collected for 10 min before injection with saline, propranolol or phentolamine and for 15 min after the experimenter had left the room (total time of disturbance <5 min). The birds were divided into two groups haphazardly. One group was injected with saline, then phentolamine, then propranolol. The other group received propranolol, then phentolamine, then saline. They were given at least 4 h for their heart rate to recover to baseline between treatments. Based on the pharmacological half-life of phentolamine in mice (50 min; Kerger *et al*., 1988) and propranolol in rats (40 min; Lemmer *et al*., 1985), we expected the drugs to be effectively cleared from the system by this point. These captivity-acclimated animals were also tested for their startle response. Their heart rate was recorded for 10 min, the door of the bird room was suddenly opened and slammed shut, and heart rate was recorded for a further 10 min.

### Experimental design

House sparrows were captured in Medford, MA, USA between 1 December 2014 and 30 June 2015. Twenty-four animals were used in the final experiments, eight in each treatment group. Immediately at capture, a series of blood samples was taken for CORT analysis (see next subsection). The birds were fitted with a harness-mounted heart rate transmitter device within 3 h of capture (see subsection heart rate transmitter harnesses). They were then transferred to individual cages in an animal facility on a 13 h light–11 h dark cycle. Birds were assigned at capture to one of three groups: saline, propranolol or phentolamine. On day 0 (the day of capture), day 1 and day 2, the birds were injected intramuscularly once per day with saline, 3 mg/kg propranolol or 3 mg/kg phentolamine. Propranolol and phentolamine were dissolved in saline at a concentration of 5 mg/ml; initial bird weight was used to calculate the injection volume for both treatments and the saline control (eg. 15 μl for a 25 g bird). Birds were held in captivity for 1 week. Heart rate was automatically sampled for 3 min every 2 h. On day 6, another series of blood samples was taken for CORT. On day 1 (before their daily injection) and day 7, the birds’ startle response was measured. Heart rate was recorded for 10 min before the startle. At time *t* = 0, the door to the room was suddenly opened and slammed closed. Heart rate was recorded for a further 10 min.

All experiments complied with Association for Assessment of Laboratory Animal Care guidelines and were approved by the Tufts Institutional Animal Care and Use Committee.

### Plasma sampling and corticosterone analysis

On days 0 and 6, a series of blood samples was taken. A baseline sample was collected within 3 min of the bird being captured or the researcher entering the bird room. The birds were held in a cloth bag for 30 min before taking a stress-induced sample. Birds were then injected intramuscularly with 1 mg/kg dexamethasone, an artificial glucocorticoid that stimulates negative feedback (Lattin *et al*., 2012). Ninety minutes after injection, a final blood sample was collected. For each sample, the alar vein was punctured and ~40 μl blood collected in a heparinized capillary tube. All blood samples were stored on ice and centrifuged at 1200***g*** for 8 min (Centrific Model 225; Fisher Scientific, Pittsburgh, PA, USA). Plasma was removed and stored at −20°C.

We determined CORT concentrations in each sample using radioimmunoassay following Wingfield *et al*. (1992). Samples were assayed in duplicate and assay values corrected for individual recoveries following extraction. Detectability was 1 ng CORT/ml plasma, the inter-assay coefficient of variation was 28% and intra-assay coefficient of variation 4%.

### Heart rate transmitter harnesses

We used the Data Sci International PhysioTel ETA-F10 model of heart rate transmitter. These transmitters measure 19 mm × 13 mm × 6 mm and weigh 1.6 g. They transmit on an AM radio frequency to a receiver plate attached to the side of the cage. They are designed to be fully implantable. Seven of the sparrows (one phentolamine, three saline and three propranolol) were implanted with transmitters following Nephew and Romero (2003). However, these small birds did not tolerate the surgery as well as the more robust European starlings, and the mortality rate was unacceptably high (50% mortality: eight of 16 birds implanted with this method died from complications with the surgery; data from the eighth surviving bird with an implanted transmitter could not be collected because of a weather emergency). Therefore, we designed a new backpack-style harness mount for the transmitters following Small *et al*. (2004).

**Figure 1:**
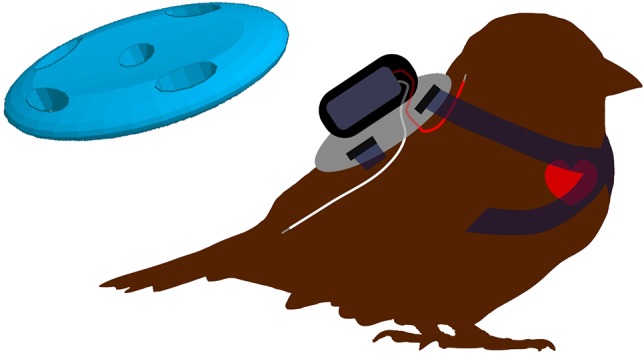
Heart rate transmitter harness design. The base of the harness is three-dimensionally printed from light plastic. The transmitter is sewn into a small fabric bag and attached to the base. Four straps (5 mm satin ribbon) are attached to the holes at the corners of the base and sewn together at the front of the animal's chest. The leads are implanted under the skin and sutured to the muscle at the back of the neck and base of the spine.

The base of the harness was three-dimensionally printed with lightweight plastic (Fig. 1). The harness base has a gently curved surface to hold the transmitter, four holes on the corners for the ribbon straps, and a hole in the centre to thread the electrodes through. The transmitter and the excess length of electrodes were sewn into a small waterproof fabric pouch, with the ends of the electrodes (~3 cm) threaded through a small hole. We sewed four lengths of 0.5-cm-wide satin ribbon (~3 cm each) to the corners of the base and secured the transmitter pouch to the ribbons with the electrodes passed through the centre hole. The ends of the ribbons were melted slightly to prevent fraying. The electrodes were then ready to be implanted.

The sparrows were anaesthetized with 4.5% isoflurane (Piramel Healthcare, Morpeth, UK) and an oxygen flow rate of 0.8 l/min using a vaporizer (Vet Equip, Livermoore, CA, USA). Once asleep, anaesthesia was maintained at ~2.5%. At the beginning of surgery, birds were injected intramuscularly with 1 mg/kg carprofen (brand name Rimadyl; Zoetis, Kalamazoo, MI, USA) as an analgesic. The top two ribbons of the harness were brought around the animal's neck and sewn in place in a V in the middle of the bird's chest. The bird was arranged on its chest, and the surgical sites were disinfected using iodine and alcohol. A small incision (5 mm) was made slightly to the left of the dorsal midline, in an area free from feather tracts. Another incision (5 mm) was made at the cervico-scapular junction. Using blunt forceps, the skin was dissected away from the muscle between the two incisions so the forceps could pass through. We then threaded the first electrode under the skin in a cranial direction. The electrode was sewn to the muscle using 4–0 synthetic monofilament suture (Ethicon, Sommerville, NJ, USA). Another surgical site was prepared on the caudo-dorsal region near the ilium. A small incision was made at the posterior site, and blunt forceps were again used to dissect the skin from the muscle between the ilium and the dorsal midline. The second electrode was then pulled through under the skin and sutured to the muscle as previously described. Wounds were closed with suture and sealed with VetBond (3M Animal Care Products, St Paul, MN, USA). The harness was then settled in place, with the electrodes completely hidden under the harness base. Exact placement of the electrodes made little difference so long as one electrode was anterior to the heart and one was posterior, so the electrical potential could be measured across the heart.

The final two ribbons of the harness were passed under the wings and sewn together, with the neck straps in the centre of the animal's chest. We monitored the birds until they recovered from the anaesthetic. Recovery from anaesthesia was uneventful. We found that a slightly tighter harness was better than one with any slack; if the straps were fairly snug, the bird was much less likely to get tangled or to be able to gain access to the electrodes or the straps. We used the harness-mounted transmitter for 17 birds in the experiments (seven phentolamine, five saline and five propranolol). However, one bird in each treatment group had a failed transmitter, so only CORT data were collected for those animals.

### Heart rate, heart rate variability and activity data collection and analysis

Heart rate was recorded automatically using DataScience's Aquisition program. Beginning in the evening of day 0 (after birds had recovered from surgery, been given their first injection and would be left undisturbed for the night) a 3 min sample was recorded every 2 h. Samples were discarded when the animals had been disturbed within 45 min of sampling (e.g. because of the caretakers, startle response sampling, or moving other animals in and out of the facility). When the program sampled heart rate, it simultaneously took a measurement of activity. The receiver plates contain within them three separate radio receivers. Any change in the relative signal strength between the three receivers is interpreted as a change in position of the animal and is recorded as ‘activity’. This is a unitless metric that is correlated with the degree of movement in the cage.

Heart rate data were analysed using the Ponemah P3 Plus program from DataSciences. This program detects the R-wave on the heart rate trace, allows for some noise detection and allows the user to inspect the data visually in order to remove inappropriate markings of R-waves. All data were carefully inspected for misplaced R-wave detection. A unitless metric of activity was recorded by the heart rate transmitters owing to changes in position relative to the receiver plate.

### Heart rate variability

Heart rate variability was calculated using Ponemah P3 Plus following the methods of Cyr *et al*. (2009). In short, a time-domain analysis was run on a clean stretch of 150–200 heart beats for each 3 min sampling window. The trace was visually inspected to ensure accurate identification of R-waves, and individual marks were adjusted as necessary. The number of marks requiring manual adjustment depended on the individual trace and ranged from 0 to 30% of R-waves. Heart rate variability is a unitless measure adjusted for heart rate, with high HRV indicating that beats are more irregular (and thus, the heart is primarily under PNS control) and low HRV indicating that they are more regular (thus, the heart is under SNS control).

### Data analysis

All statistical analyses were conducted in R version 3.1.3 (R Core Team, 2013). Linear mixed-effects models were constructed using the ‘lmer’ function in the lme4 package of R (Bates *et al*., 2015). Bird identity was included as a random effect in all analyses. We then used the ‘Anova’ function in the car package (Fox and Weisberg, 2011) to calculate Type II Wald *F*-tests with Kenward–Roger adjusted degrees of freedom. We followed this with Tukey's multiple comparison test if warranted, using the ‘glht’ function from the multcomp package (Hothorn *et al*., 2008). An α-value of *P* < 0.05 was used to determine significance. To test for normality, we used the ‘qqp’ function from the car package in R (Fox and Weisberg, 2011), which generates theoretical quantile–quantile (q-q) plots to compare our data with a normal distribution, with 95% confidence interval lines. We considered our data to be normally distributed when the majority of observations fell within the expected range.

To test for the acute effect of propranolol, phentolamine or saline injection on heart rate, we measured integrated heart rate for 15 min post-injection. (This is the area under the curve, representing the total number of additional heart beats above baseline that the bird experienced.) We tested the effect of treatment on integrated heart rate. This was followed by a Tukey's multiple comparison test on finding significance. We tested for an effect of transmitter surgery (harness mounted vs. implanted) on resting heart rate, activity or HRV (day and night analysed separately). We also confirmed that transmitter surgery type had no effect on baseline CORT, stress-induced CORT or the strength of negative feedback after dexamethasone injection.

Baseline CORT, stress-induced CORT and strength of negative feedback were analysed in separate models. Baseline CORT was very skewed because many samples were below the limit of detection of the assay. We used non-parametric Kruskal–Wallis tests to look for differences between CORT concentration at day 0 vs. day 6 (treatment groups analysed together and separately). We also tested for differences between treatment groups at capture and after 1 week. The strength of negative feedback in the hypothalamic–pituitary–adrenal (HPA) axis was calculated as the percentage decrease from stress-induced CORT 90 min after a dexamethasone injection. We confirmed that stress-induced CORT and strength of negative feedback were normally distributed by comparison with theoretical q-q plots for normality. We tested for the effect of treatment, experiment day and their interaction on stress-induced CORT and negative feedback strength.

Resting heart rate, activity and heart rate variability were all analysed in the same way. For visual simplicity, we averaged values across each day and night for each bird and used these averages to represent the data graphically. For the analysis, however, we used all sampling points. First, we confirmed that there were circadian patterns in these variables by looking for an effect of day vs. night. We analysed daytime and night-time separately on finding circadian rhythms in all variables. We confirmed that the data were normally distributed by comparison with theoretical q-q plots. Some data were not normally distributed and were transformed as follows. Daytime heart rate data were negatively skewed, so were normalized by squaring. The transformed daytime heart rate data were still non-normal, with a kurtosis of −1.13 (a ‘flattened’ distribution). However, linear mixed models are robust against kurtosis violations, even when they are sensitive to skewness violations (Arnau *et al*., 2013). Heart rate variability and daytime activity data were positively skewed and were normalized by natural logarithmic transformation. After transformation, HRV and daytime activity data were slightly negatively skewed (respectively, −0.25 and −0.41). With this degree of skew, our analysis method is robust for the HRV analysis, but there is an increased risk of type 1 error for the activity data (Arnau *et al*., 2013). For each day or night, each bird had up to six measurements (taken every 2 h). There was very little activity at night (635 of 799 night-time data points had activity ≤2), so these data were not analysed statistically. Final models included experiment day, treatment and their interaction. We then compared newly captured birds with birds held in captivity for 1 month, combining all treatments when there was no treatment effect.

We analysed two variables for the startle response: maximal heart rate after the startle and integrated heart rate for the first 10 min after the startle (area under the curve). Both maximal heart rate and integrated heart rate were normally distributed as confirmed by comparison with theoretical q-q plots for normality. Linear mixed models for the effects of experiment day, treatment and their interaction were run on these variables. We also compared maximal and integrated heart rate at day 1, day 7 and after 1 month (all treatments combined), followed by Tukey's *post hoc* comparisons as warranted.

## Results

### Drug and harness-mounted transmitter validations

**Figure 2:**
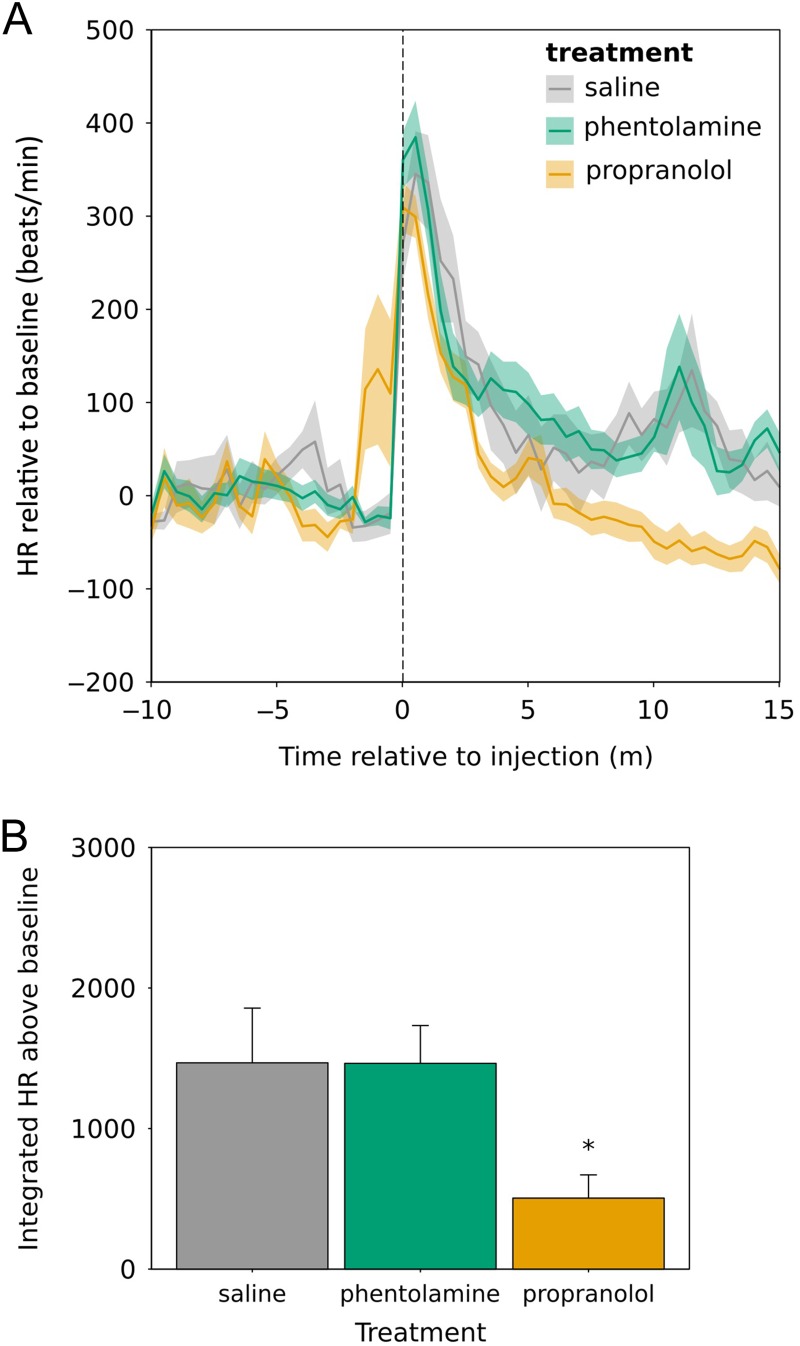
Injection of propranolol results in reduced heart rate (HR) relative to saline or phentolamine injection. (**A**) Heart rate relative to baseline. The shaded area indicates one standard error around the mean. Heart rate was recorded before disturbance and after the experimenter had left the room at *t* = 0. (**B**) Integrated heart rate relative to baseline from 0–15 min (i.e. area under the curve). Error bars represent means + SEM. *Significant difference (*P* < 0.05) relative to saline and phentolamine.

Propranolol, phentolamine and saline were injected into eight animals that had been living in captivity for >28 days. Heart rate was measured for 10 min before the disturbance and 15 min after the experimenter had left the room after injecting all birds. The injection procedure caused an increase in heart rate in all animals (Fig. 2A). Integrated heart rate after injection was significantly different between treatment groups (*F*_2,13.2_ = 6.74, *P* = 0.01; Fig. 2B). Integrated heart rate was lower after propranolol treatment than after phentolamine or saline (Tukey's *post hoc* analysis: propranolol vs. phentolamine, *z* = 3.22, *P* = 0.004; propranolol vs. saline, *z* = −3.10, *P* = 0.005; phentolamine vs. saline, *z* = 0.02, *P* = 1).

There was no difference in daytime or night-time heart rate between implanted vs. harness-mounted heart rate transmitters (day, *F*_1,20.2_ = 0.52, *P* = 0.5; night, *F*_1,20.2_ = 1.73, *P* = 0.20). There was no difference in daytime or night-time HRV between implanted vs. harness-mounted transmitters (day, *F*_1,20.1_ = 0.02, *P* = 0.9; night, *F*_1,20.3_ = 0.33, *P* = 0.6). One week post-capture, there was no difference in baseline CORT (Kruskal–Wallis, χ^2^ = 0.78, d.f. = 1, *P* = 0.4), stress-induced CORT (*F*_1,22_ = 1.03, *P* = 0.32) or the strength of negative feedback after a dexamethasone challenge (*F*_1,22_ = 0.71, *P* = 0.4). Daytime activity was higher with the harness-mounted transmitters compared with implanted transmitters (*F*_1,19.9_ = 4.18, *P* = 0.05). However, given the lack of difference in all other physiological variables and the small number of implanted transmitters, no further distinction was made between transmitter placement, and data were combined for all further analyses.

### Weight

During the first week of captivity, 87.5% (21 of 24) of the animals lost weight. Weight loss was not significantly different between treatment groups (*F*_2,21_ = 1.91, *P* = 0.17). Birds lost on average 11% of their starting mass over the course of 1 week.

### Corticosterone responses

**Figure 3:**
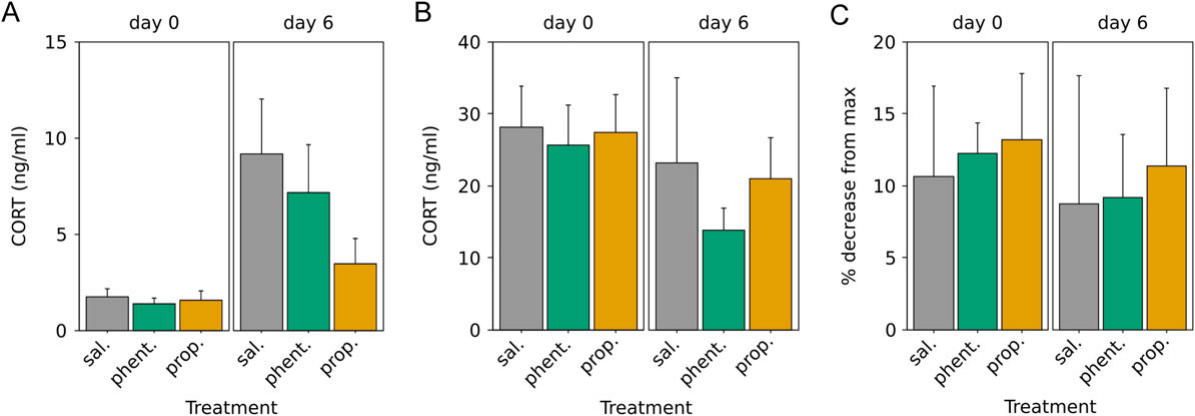
Corticosterone (CORT) response to stress at capture and after 1 week. (**A**) Baseline CORT concentrations taken within 3 min of capture or disturbance. (**B**) Stress-induced CORT was taken after the birds were held for 30 min in a cloth bag. (**C**) Strength of negative feedback is calculated as the percentage decrease in CORT concentration from the stress-induced sample 90 min after injection with dexamethasone. Error bars represent means + SEM.

Baseline CORT was low, and many samples were below the limit of detection (28% of samples), so non-parametric Kruskal–Wallis tests were used. Baseline CORT was significantly higher at day 6 compared with day 0 when treatments were combined (Kruskal–Wallis, χ^2^ = 10.87, d.f. = 1, *P* = 0.001; Fig. 3A). There was no effect of treatment on day 0 (χ^2^ = 2.87, d.f. = 2, *P* = 0.2) or day 6 (χ^2^ = 3.36, d.f. = 2, *P* = 0.2). We also looked for a difference between day 0 and day 6 in baseline CORT in each treatment group separately. Baseline CORT increased between day 0 and day 6 in saline- and phentolamine-treated birds (respectively, χ^2^ = 6.55, d.f. = 1, *P* = 0.01; χ^2^ = 4.98, d.f. = 1, *P* = 0.03), but not in propranolol-treated birds (χ^2^ = 1.38, d.f. = 1, *P* = 0.2).

Stress-induced CORT was not affected by experiment day, treatment or their interaction (experiment day, *F*_2,21_ = 2.23, *P* = 0.2; treatment, *F*_2,21_ = 0.39, *P* = 0.7; interaction, *F*_2,21_ = 0.16, *P* = 0.9; Fig. 3B). There was also no effect of experiment day, treatment or their interaction on the strength of negative feedback after dexamethasone (experiment day, *F*_1,21_ = 0.24, *P* = 0.6; treatment, *F*_2,21_ = 0.11, *P* = 0.9; interaction, *F*_2,21_ = 0.01, *P* = 1; Fig. 3C).

### Heart rate

**Figure 4:**
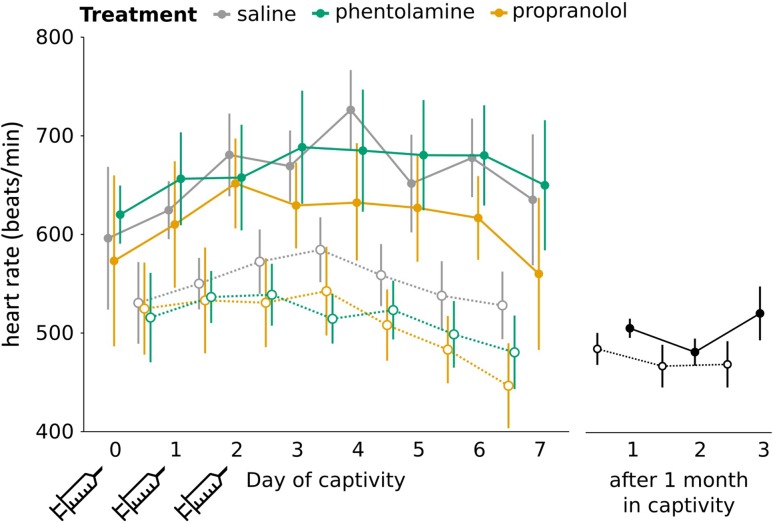
Resting heart rate over the course of the first week of captivity. Syringes indicate days of saline, propranolol or phentolamine treatment. Daytime heart rate (continuous lines and filled circles) is higher than night-time heart rate (dotted lines and open circles) during the first week and after 1 month in captivity. Daytime heart rate is higher in newly captive birds than in birds kept in captivity for 1 month. There were no differences between treatment groups. Error bars represent means ± SE.

During the first week of captivity, resting heart rate was higher during the day than at night (*F*_1,1390.3_ = 683.0, *P* < 0.00001; Fig. 4). In birds that had been held in captivity for 1 month, we also saw a circadian pattern in resting heart rate (*F*_1,455.1_ = 15.86, *P* < 0.0001; Fig. 4). We therefore analysed daytime and night-time heart rate separately. Daytime heart rate was negatively skewed, so was squared for analysis. Daytime heart rate did not change over the course of the first 10 days and did not differ by treatment (treatment, *F*_2,25.0_ = 0.07, *P* = 0.9; experiment day, *F*_1,584.2_ = 0.31, *P* = 0.6; interaction, *F*_2,584.5_ = 0.79, *P* = 0.5). We compared daytime heart rate between newly captive birds (all treatments combined) and birds held in captivity for 1 month. Heart rate was significantly higher in newly captured birds compared with birds held in captivity for 1 week (*F*_1,28_ = 13.77, *P* = 0.001). Night-time heart rate decreased over the course of the first week of captivity, but there was no treatment effect and no interaction effect (experiment day, *F*_1,764.9_ = 4.51, *P* = 0.03; treatment, *F*_2,21.3_ = 0.17, *P* = 0.84; interaction, *F*_2,764.9_ = 1.0, *P* = 0.4). There was no difference in night-time heart rate between the newly captive birds (all treatment groups combined) and birds that had been in captivity for 1 month (*F*_1,28_ = 2.74, *P* = 0.1).

### Activity

**Figure 5:**
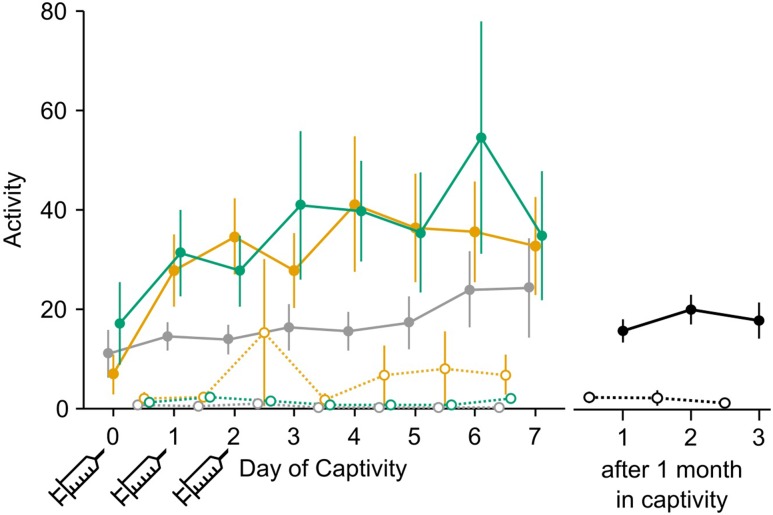
Activity levels (a unitless metric) are greater during the day than at night. Syringes indicate days of saline, propranolol or phentolamine treatment. Daytime heart rate (continuous lines and filled circles) is higher than night-time heart rate (dotted lines and open circles) during the first week and after 1 month in captivity. Daytime heart rate increases over the course of the first week of captivity. Error bars indicate means ± SEM.

Activity is a unitless metric derived from the heart rate transmitters. We found a strong circadian rhythm in activity, with daytime activity much higher than night-time (*F*_1,1357.2_ = 1630.3, *P* < 0.00001; Fig. 5). This same pattern was seen in birds held in captivity for 1 month (*F*_1,449.1_ = 466.15, *P* < 0.00001; Fig. 5). We analysed daytime and night-time activity separately. Daytime activity data were positively skewed and so were logarithmically transformed for analysis. During the day, there was no effect of experiment day, treatment or their interaction (experiment day, *F*_1,550.2_ = 1.11, *P* = 0.3; treatment, *F*_2,30.4_ = 1.01, *P* = 0.4; interaction, *F*_2,551.7_ = 0.01, *P* = 1). There was no difference in activity level between newly captured birds (all treatments combined) and 1 month captives (*F*_1,28_ = 0.03, *P* = 0.9). Night-time data were skewed such that they could not be normalized. There was little activity at night (79% of activity values measured were <2), and there was no apparent pattern with treatment or day.

### Heart rate variability

**Figure 6:**
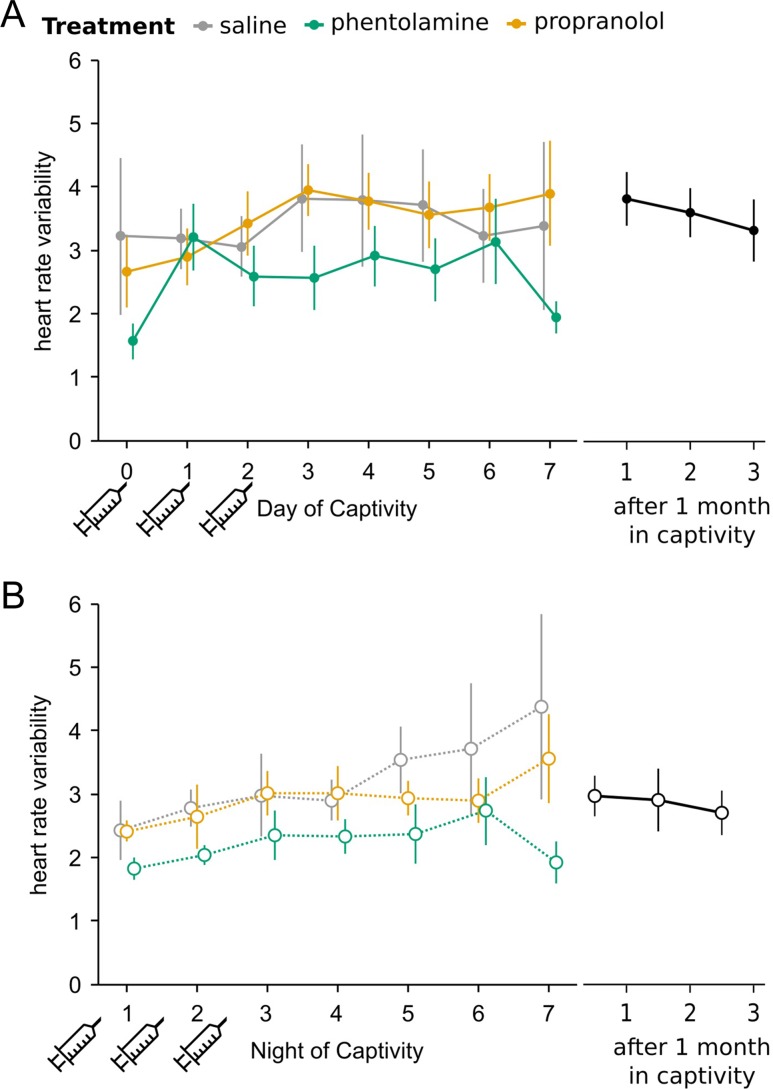
Heart rate variability (HRV) during the first week of captivity. Heart rate variability is higher during the day than at night in recent captives and after 1 month captivity. (**A**) Daytime HRV increases over the first week of captivity. (**B**) Night-time HRV increases over the course of the experiment in saline- but not propranolol- or phentolamine-treated birds. Syringes indicate days of saline, propranolol or phentolamine treatment. Points represent means ± SE.

Heart rate variability data were positively skewed and therefore logarithmically transformed before analysis. During the first week of captivity, HRV was higher during the day than at night (*F*_1,1322.9_ = 33.35, *P* < 0.00001). This pattern was maintained in birds held in captivity for 1 month (*F*_1,448_ = 57.12, *P* < 0.00001). We therefore analysed daytime and night-time HRV separately. There was an effect of experiment day on daytime HRV, as well as an interaction effect, but no effect of treatment (experiment day, *F*_1,526.5_ = 6.66, *P* = 0.01; treatment, *F*_2,25.9_ = 0.17, *P* = 0.8; interaction, *F*_2,527.6_ = 3.87, *P* = 0.02; Fig. 6A). As a result of the interaction effect, we looked for an effect of experiment day on each treatment separately. Daytime HRV significantly increased over time in the saline and propranolol groups (saline, *F*_1,207.2_ = 5.74, *P* = 0.02; propranolol, *F*_1,144_ = 3.77, *P* = 0.05). However, there was no effect of experimental day in the phentolamine group (*F*_1,176.4_ = 1.37, *P* = 0.2). We compared daytime HRV during the first week of captivity (all treatment groups combined) with 1 month captives and found no difference (*F*_1,27.9_ = 1.12, *P* = 0.3).

At night, HRV increased over time (*F*_1,756.2_ = 61.65, *P* < 0.00001; Fig. 6B). There was no effect of treatment, but there was a significant interaction between treatment and experiment day (treatment, *F*_2,23.6_ = 1.03, *P* = 0.4; interaction, *F*_2,755.9_ = 10.97, *P* < 0.00001). As a result of the interaction effect, we tested the effect of experiment day on each treatment group separately. Night-time HRV significantly increased over the first week of captivity in the saline and phentolamine groups (saline, *F*_1,275.9_ = 56.1, *P* < 0.00001; phentolamine, *F*_1,263.1_ = 4.02, *P* = 0.05). There was a marginally significant trend towards increasing HRV in the propranolol group (*F*_1,216.8_ = 3.72, *P* = 0.06). We compared night-time HRV during the first week of captivity (all treatment groups combined) with 1 month captives and found no difference (*F*_1,28_ = 0.16, *P* = 0.7).

### Startle response

**Figure 7:**
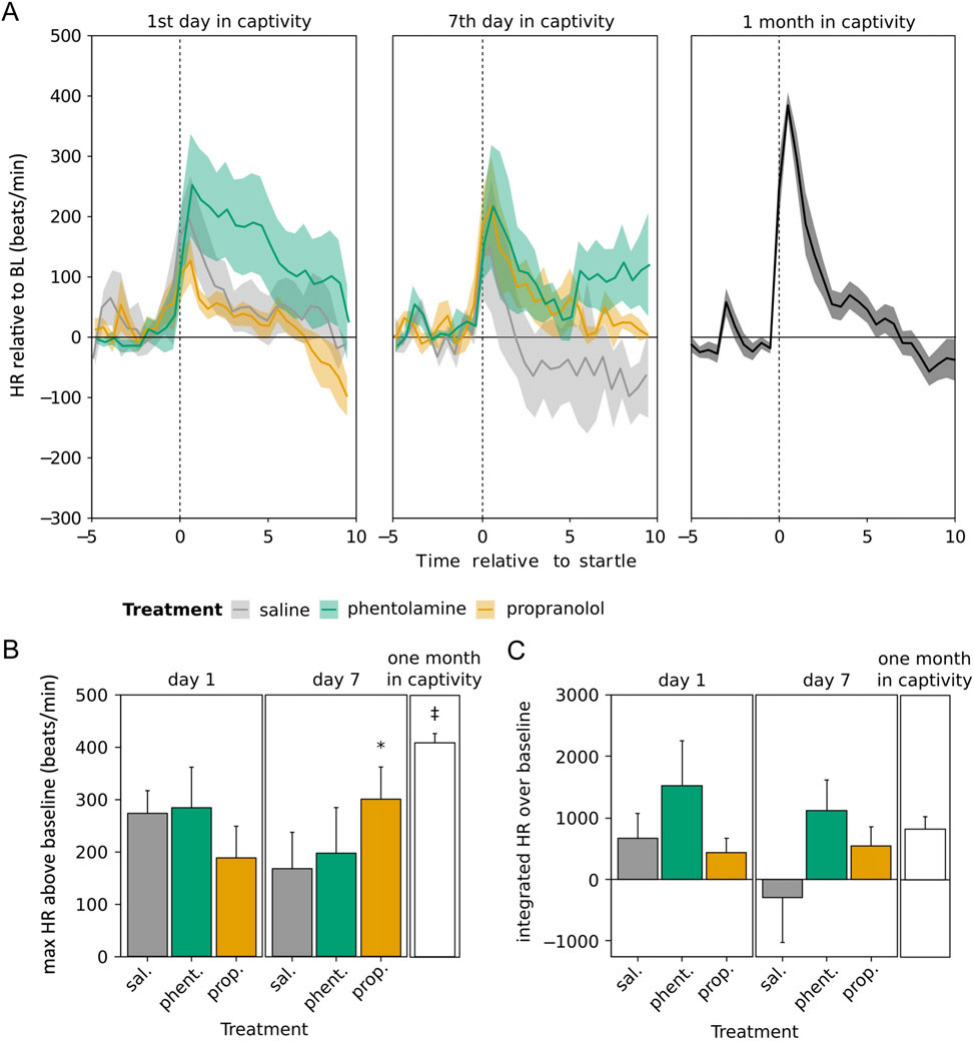
(**A**) Startle tests on the first and seventh day of captivity and after 1 month. Shaded area indicates one standard error around the mean. (**B**) Maximal heart rate (HR) after the startle. *P < 0.05 compared with first day of captivity; ‡*P* < 0.05 compared with day 1 and day 7. (**C**) Integrated heart rate relative to baseline (BL) from *t* = 0 to 10. Error bars indicate means ± SEM.

A startle response was measured after 1 day in captivity (one injection had been received 18–24 h earlier) and after 7 days in captivity (three injections had been received, with the last one being 4 days before; Fig. 7A). There was no overall difference in maximal heart rate between day 1 and day 7, and no effect of treatment (experiment day, *F*_1,15.3_ = 0.31, *P* = 0.6; treatment, *F*_2,17.4_ = 0.03, *P* = 1; Fig. 7B). However, there was a marginally significant interaction effect (*F*_2,15.2_ = 3.00, *P* = 0.08). We therefore compared day 1 with day 7 for each treatment group separately. We found that propranolol-treated birds had significantly higher maximal heart rate at day 7 compared with day 1 (*F*_1,5.3_ = 10.49, *P* = 0.02), but maximal heart rate did not change between day 1 and day 7 in saline- or phentolamine-treated birds (saline, *F*_1,4.3_ = 1.51, *P* = 0.28; phentolamine, *F*_1,5.4_ = 0.86, *P* = 0.39). We then compared maximal heart rate at day 1, day 7 and after 1 month (treatment groups combined) and found no effect of duration in captivity (*F*_2,29.0_ = 3.24, *P* = 0.05). Maximal heart rate at 1 month was significantly higher than at day 1 or day 7 (Tukey's *post hoc* analysis: 1 month vs. day 1, *z* = 2.31, *P* = 0.05; 1 month vs. day 7, *z* = 2.51, *P* = 0.03; day 1 vs. day 7, *z* = −0.33, *P* = 0.9).

Integrated heart rate was calculated for the 10 min following the startle. There was no difference in integrated heart rate on day 1 vs. day 7, no effect of treatment and no interaction effect (experiment day, *F*_1,13.7_ = 1.33, *P* = 0.3; treatment, *F*_2,17.1_ = 1.72, *P* = 0.2; interaction, *F*_2,13.6_ = 0.91, *P* = 0.4; Fig. 7C). We compared integrated heart rate at day 1, day 7 and after 1 month (all treatment groups combined) and found no effect (*F*_2,26.7_ = 0.43, *P* = 0.66).

## Discussion

### Effects of chronic stress on corticosterone

The direction, intensity and timing of the changes caused by chronic stress depends on the species and the type of stressor (Dickens and Romero, 2013). However, a change in glucocorticoid regulation is typically seen during chronic stress. Glucocorticoid concentrations have been demonstrated to change when wild animals are first brought into captivity in mammals (Terio *et al*., 2004; Cabezas *et al*., 2007; Franceschini *et al*., 2008), birds (Dickens *et al*., 2009; Adams *et al*., 2011; Lattin *et al*., 2012), reptiles (Jones and Bell, 2004) and amphibians (Narayan *et al*., 2011). The effects of captivity on the HPA axis in house sparrows at different times of year was previously reported by Lattin *et al*. (2012). They found that captivity affects CORT production differently during different life-history stages, but during most or all seasons, birds lost weight, baseline CORT increased, and the strength of negative feedback in the CORT response increased.

Consistent with the findings of Lattin *et al*. (2012), the birds in our study lost weight, and we saw an increase in baseline CORT concentrations in saline- and phentolamine-treated birds. Following propranolol treatment, baseline CORT did not increase, so one symptom of chronic stress was reduced. Propranolol-treated birds may have had a less dysregulated HPA axis as a result of their treatment. The pituitary normally releases ACTH when it receives a signal of corticotrophin-releasing factor (CRF) from the hypothalamus. However, CRF is not the only molecule that causes ACTH production; among other secretagogues, E and NE can both stimulate ACTH production (Mezey *et al*., 1983; Bugajski *et al*., 1995). During chronic stress, other ACTH secretagogues may be as important as CRF, because CRF production would be shut down by negative feedback of CORT on the hypothalamus. If E and NE are blocked from acting at the pituitary, less ACTH and therefore less CORT would be secreted. During the first few days of a chronic stressor, this reduction of CORT production, even if temporary, may help to prevent or at least delay the dysregulation of the HPA axis that leads to higher baseline CORT. Propranolol, but not phentolamine, prevents the increase in baseline CORT. This suggests that β-receptors in the pituitary are responsible for stimulating ACTH production, as has been found in rats (Mezey *et al*., 1983; Bugajski *et al*., 1995) and chickens (Rees *et al*., 1985). However, the potential of propranolol to reduce HPA symptoms of chronic stress should not be overstated, as there was no overall treatment effect on baseline CORT on day 6.

### Effects of chronic stress on the sympathetic nervous system in house sparrows

The effects of chronic captivity stress on heart rate in house sparrows has not been previously documented. Using our heart rate transmitter equipment, it is not possible to obtain heart rate data from a bird that is not currently in captivity; the bird must remain within ~0.3 m of the receiver plate. We therefore compared heart rate data from newly captured birds with data from birds that had been held in captivity for >1 month; these birds have presumably acclimated to the conditions of captivity. However, we have no way of knowing whether this represents a physiology similar to wild birds, or if captivity permanently alters the physiology of these animals.

β-Blockers typically reduce the heart rate response to stressors in both mammals (Ballard-Croft and Horton, 2002) and birds (Cyr *et al*., 2009). In our validation tests, propranolol caused a decrease in integrated heart rate over the 15 min following injection compared with phentolamine or saline. Phentolamine did not cause an acute change in HR. It is important to recognize that the treatments we administered were very transient. Propranolol has a pharmacological half-life of ~40 min in rats (Lemmer *et al*., 1985). The half-life of phentolamine in mice is ~50 min (Kerger *et al*., 1988). Therefore, a 3 day treatment of these drugs administered once per day is a very mild intervention, probably resulting in moderately decreased SNS activity for at most a few hours per day.

During the first week of captivity, newly captured birds had much higher daytime heart rates than the 1 month captives. This suggests that sometime between the first week and 1 month of captivity, HR decreases. In a previous study on European starlings newly brought into captivity, HR was elevated compared with long-term captives only for the first 24 h (Dickens and Romero, 2009). These data cannot be explained by changes in the animals’ activity levels, as there was no difference in activity between newly captive birds and 1 month captives. We cannot isolate which factor or factors cause the high heart rate in the first week, but the birds presumably can adapt gradually to captive conditions.

Heart rate variability was not different when comparing recent captives with 1 month captives. This is in contrast to previous work in the European starling, where HRV was low during the first 48 h of captivity, gradually increasing towards the same level as long-term captives (Dickens and Romero, 2009). Although we saw no difference in HRV between newly captured birds and 1 month captives, HRV did increase over the course of the first week during both day and night. This indicates increasing PNS or decreasing SNS activity over time, as we would expect to see in animals that are acclimating to the conditions of captivity. Low HRV over a short time scale has been associated with cardiac disease in humans (Stauss, 2003) and has been used as an indicator of poor welfare in other animals (von Borell *et al*., 2007). HRV can provide more information about the state of an animal than heart rate alone. For example, layer hens from high-feather-plucking lines had only a slight elevation in resting heart rate compared with low-feather-plucking lines, but they showed substantially reduced HRV (Korte *et al*., 1999). Therefore, we expect that the health of the animals increases over the course of the first week. Even though heart rate remains high, increasing HRV may mean that the birds experience less stress as they acclimate to captivity.

The circadian pattern in HRV, with higher HRV during the day, which was present in both recent captives and long-term captives, was unexpected. This indicates higher parasympathetic activity and lower sympathetic activity during the day than at night, which is counter-intuitive. A possible explanation may be that daytime HRV does not represent a resting animal. If the birds were moving around during the sampling period, their heart rate might be somewhat more irregular because of movement, not because of parasympathetic control. The higher variability would then be explained by greater activity during the day, rather than by the natural rhythm of the PNS. Night-time HRV is more likely to represent resting heart rate, without the influence of activity, because activity at night was generally low.

Phentolamine-treated birds differed from saline-treated control birds in how their HRV changed over time. In saline-treated birds, daytime HRV increased over the course of 7 days. This shifting to more parasympathetic control suggests that the birds were acclimating to captivity. In phentolamine-treated birds, HRV did not increase, but stayed low throughout the week. Therefore, treating with an α-blocker led to higher SNS activation/lower PNS activation over 1 week. As a consequence, phentolamine treatment possibly resulted in a less favourable outcome than control treatment, although the pattern is not statistically significant at night, when HRV values are probably more reliable.

The continued high sympathetic activity may be explained by the role of α-receptors in the negative feedback of NE. Epinephrine and NE are released as hormones from the adrenal medulla. However, they are also released as neurotransmitters throughout the nervous system. Although phentolamine cannot cross the blood–brain barrier, it can act on nerves in the periphery (Langer, 1980). α-Receptors are involved in an autocrine feedback loop on presynaptic neurons for the regulation of NE both in the brain and in peripheral nerves. During stress, NE is released into the synapse, where it binds to α_2A_-receptors on the presynaptic neuron to shut down further NE production (Langer, 1980; Callado and Stamford, 1999). By blocking the α-receptors with phentolamine, the feedback loop is broken and more NE signalling will occur. This temporary disturbance of NE regulation appears to have long-term consequences. Even long after phentolamine treatment has stopped, SNS activity remains high. During chronic stress, tree shrews upregulated the α_2A_-receptors in their brain (Fluegge *et al*., 2003), increasing the sensitivity of the feedback loop. However, if phentolamine disrupts this regulatory signalling, the upregulation of α_2A_-receptors might not occur and high NE concentrations may continue.

### Startle response

In a previous study on European starlings, a striking difference between newly captured birds and long-term captives was the severe reduction, almost elimination, of the cardiac response to startle (Dickens and Romero, 2009). Even after 10 days of captivity, the birds showed almost no heart rate reaction to a sudden loud noise. A potentially diminished startle response during the transition to captivity has been reported in other animals. Newly captured bighorn sheep had lower plasma concentrations of E and NE during an acute stressor than sheep raised in captivity (Coburn *et al*., 2010). Captive harbor porpoises had lower plasma E and NE after being netted and sampled than free-living porpoises (Siebert *et al*., 2011). In house sparrows in the present study, the startle response was somewhat reduced but not to the same degree as in European starlings. There was no statistically significant difference in integrated heart rate between recent captives and 1 month captives, and no difference between treatment groups. However, maximal heart rate relative to baseline during a startle response was higher in 1 month captives compared with recently captured animals. This may have been attributable to the higher baseline heart rate in newly captured birds; the heart may not be able to beat any faster. Propranolol treatment may have affected maximal heart rate; propranolol-treated birds had a higher maximal heart rate on day 7 than they did on day 1. The loss of the startle response could profoundly impact the well-being of birds. Although the detrimental effect of an impaired startle response on survival has not, to our knowledge, been tested directly, a study in wild and domesticated lines of Atlantic salmon showed that a reduced cardiac response to a simulated predator was correlated with reduced escape behaviours (Johnsson *et al*., 2001). If birds are brought into captivity for translocation, an impaired startle response at release could potentially reduce their ability to escape from predators (for a review on the effects of stress on translocation success, see Dickens *et al*., 2010). Therefore, retaining a healthy startle response, perhaps by the use of propranolol, could be important for captive birds. An experimental evaluation of the connection between the startle response and survival would be an exciting avenue for future research.

### Conclusion

Treatment with propranolol appeared to have a net positive effect by attenuating the development of some chronic stress symptoms in house sparrows during the first week of captivity. Propranolol prevented the increase in baseline CORT caused by captivity. Propranolol treatment also caused an increase in maximal startle response over the first week of captivity, although it was still not as high as 1 month captives. Phentolamine treatment did not have an effect on any chronic stress symptoms we measured. Neither propranolol treatment nor phentolamine treatment significantly affected heart rate during the first week of captivity, although phentolamine may have an adverse effect in keeping HRV low and PNS activity high.

## Funding

This work was supported by National Science Foundation grant IOS-1048529 awarded to L.M.R.

## References

[cow049C1] Adams NJ, Farnworth MJ, Rickett J, Parker KA, Cockrem JF (2011) Behavioural and corticosterone responses to capture and confinement of wild blackbirds (*Turdus merula*). Appl Anim Behav Sci 134: 246–255.

[cow049C2] Ahlquist RP (1948) A study of the adrenotropic receptors. Am J Physiol 153: 586–600. 18882199 10.1152/ajplegacy.1948.153.3.586

[cow049C3] Arnau J, Bendayan R, Blanca MJ, Bono R (2013) The effect of skewness and kurtosis on the robustness of linear mixed models. Behav Res Methods 45: 873–879. 23299397 10.3758/s13428-012-0306-x

[cow049C4] Ballard-Croft C, Horton JW (2002) Sympathoadrenal modulation of stress-activated signaling in burn trauma. J Burn Care Res 23: 172–182. 10.1097/00004630-200205000-00006 12032367

[cow049C5] Bates D, Maechler M, Bolker B, Walker S (2015) Fitting linear mixed-effects models using lme4. J Stat Softw 67: 1–48.

[cow049C6] Bugajski J, Gadekmichalska A, Ołowska A, Borycz J, Głód R, Bugajski A (1995) Adrenergic regulation of the hypothalamic–pituitary–adrenal axis under basal and social stress conditions. J Physiol Pharmacol 46: 297–312. 8527811

[cow049C7] Cabezas S, Blas J, Marchant TA, Moreno S (2007) Physiological stress levels predict survival probabilities in wild rabbits. Horm Behav 51: 313–320. 17258747 10.1016/j.yhbeh.2006.11.004

[cow049C8] Callado L, Stamford J (1999) α_2A_- but not α_2B/C_-adrenoceptors modulate noradrenaline release in rat locus coeruleus: voltammetric data. Eur J Pharmacol 366: 35–39. 10064149 10.1016/s0014-2999(98)00889-9

[cow049C9] Coburn S, Salman M, Rhyan J, Keefe T, McCollum M (2010) Comparison of endocrine response to stress between captive-raised and wild-caught bighorn sheep. J Wildl Manage 74: 532–538.

[cow049C10] Cyr N, Dickens M, Romero LM (2009) Heart rate and heart rate variability responses to acute and chronic stress in a wild-caught passerine bird. Physiol Biochem Zool 82: 332–344. 19115847 10.1086/589839

[cow049C11] Dickens MJ, Romero LM (2009) Wild European starlings (*Sturnus vulgaris*) adjust to captivity with sustained sympathetic nervous system drive and a reduced fight-or-flight response. Physiol Biochem Zool 82: 603–610. 19642947 10.1086/603633

[cow049C12] Dickens MJ, Romero LM (2013) A consensus endocrine profile for chronically stressed wild animals does not exist. Gen Comp Endocrinol 191: 177–189. 23816765 10.1016/j.ygcen.2013.06.014

[cow049C13] Dickens MJ, Nephew BC, Romero LM (2006) Captive European starlings (*Sturnus vulgaris*) in breeding condition show an increased cardiovascular stress response to intruders. Physiol Biochem Zool 79: 937–943. 16927240 10.1086/506007

[cow049C14] Dickens MJ, Earle KA, Romero LM (2009) Initial transference of wild birds to captivity alters stress physiology. Gen Comp Endocrinol 160: 76–83. 19026651 10.1016/j.ygcen.2008.10.023

[cow049C15] Dickens MJ, Delahanty DJ, Romero LM (2010) Stress: an inevitable component of animal translocation. Biol Conserv 143: 1329–1341.

[cow049C45] Fluegge G, van Kampen M, Meyer H, Fuchs E (2003) Alpha2A and alpha2C-adrenoceptor regulation in the brain: Alpha2A changes persist after chronic stress. Eur J Neurosci 17: 917–928. 12653968 10.1046/j.1460-9568.2003.02510.x

[cow049C16] Fox J, Weisberg S (2011) An {R} Companion to Applied Regression. Sage, Thousand Oaks, CA.

[cow049C17] Franceschini MD, Rubenstein DI, Low B, Romero LM, (2008) Fecal glucocorticoid metabolite analysis as an indicator of stress during translocation and acclimation in an endangered large mammal, the Grevy's zebra. Anim Conserv 11: 263–269.

[cow049C18] Hothorn T, Bretz F, Westfall P (2008) Simultaneous inference in general parametric models. Biometrical J 50: 346–363. 10.1002/bimj.200810425 18481363

[cow049C19] Johnsson JI, Höjesjö J, Fleming IA (2001) Behavioural and heart rate responses to predation risk in wild and domesticated Atlantic salmon. Can J Fish Aquat Sci 58: 788–794.

[cow049C20] Jones S, Bell K (2004) Plasma corticosterone concentrations in males of the skink *Egernia whitii* during acute and chronic confinement, and over a diel period. Comp Biochem Physiol A Mol Integr Physiol 137: 105–113. 14720596 10.1016/s1095-6433(03)00267-8

[cow049C21] Kerger BD, James RC, Roberts SM (1988) An assay for phentolamine using high performance liquid chromatography with electrochemical detection. Anal Biochem 170: 145–151. 3389507 10.1016/0003-2697(88)90102-9

[cow049C22] Korte S, Ruesink W, Blokhuis H (1999) Heart rate variability during manual restraint in chicks from high- and low-feather pecking lines of laying hens. Physiol Behav 65: 649–652. 10073462 10.1016/s0031-9384(98)00206-6

[cow049C23] Langer SZ (1980) Presynaptic regulation of the release of catecholamines. Pharmacol Rev 32: 337–362. 6267618

[cow049C24] Lattin CR, Bauer CM, de Bruijn R, Romero LM (2012) Hypothalamus–pituitary–adrenal axis activity and the subsequent response to chronic stress differ depending upon life history stage. Gen Comp Endocrinol 178: 494–501. 22841762 10.1016/j.ygcen.2012.07.013

[cow049C25] Lemmer B, Winkler H, Ohm T, Fink M (1985) Chronopharmacokinetics of beta-receptor blocking drugs of different lipophilicity (propranolol, metoprolol, sotalol, atenolol) in plasma and tissues after single and multiple dosing in the rat. Naunyn Schmiedebergs Arch Pharmacol 330: 42–49. 2864639 10.1007/BF00586708

[cow049C26] Mahata S, Ghosh A (1991) Role of splanchnic nerve on steroid-hormone-induced alteration of adrenomedullary catecholamines in untreated and reserpinized pigeon. J Comp Physiol B 161: 598–601. 1783691 10.1007/BF00260750

[cow049C27] Mezey E, Reisine TD, Palkovits M, Brownstein MJ, Axelrod J (1983) Direct stimulation of β_2_-adrenergic receptors in rat anterior pituitary induces the release of adrenocorticotropin *in vivo*. Proc Natl Acad Sci USA 80: 6728–6731. 6314339 10.1073/pnas.80.21.6728 PMC391244

[cow049C28] Minneman K, Pittman R, Molinoff P (1981) β-Adrenergic-receptor subtypes: properties, distribution, and regulation. Annu Rev Neurosci 4: 419–461. 6111980 10.1146/annurev.ne.04.030181.002223

[cow049C29] Morgan KN, Tromborg CT (2007) Sources of stress in captivity. Appl Anim Behav Sci 102: 262–302.

[cow049C30] Narayan EJ, Cockrem JF, Hero J (2011) Urinary corticosterone metabolite responses to capture and captivity in the cane toad (*Rhinella marina*). Gen Comp Endocrinol 173: 371–377. 21756910 10.1016/j.ygcen.2011.06.015

[cow049C31] Nephew B, Romero LM (2003) Behavioral, physiological, and endocrine responses of starlings to acute increases in density. Horm Behav 44: 222–232. 14609544 10.1016/j.yhbeh.2003.06.002

[cow049C32] Perini R, Veicsteinas A (2003) Heart rate variability and autonomic activity at rest and during exercise in various physiological conditions. Eur J Appl Physiol 90: 317–325. 13680241 10.1007/s00421-003-0953-9

[cow049C33] R Core Team (2013) *R: a Language and Environment for Statistical Computing*. Version 3.1.3. R Foundation for Statistical Computing, Vienna.

[cow049C34] Rees A, Harvey S, Phillips J (1985) Adrenergic stimulation of adrenocortical secretion in immature fowl. Comp Biochem Phys C 81: 387–389. 10.1016/0742-8413(85)90024-6 2861958

[cow049C35] Rich EL, Romero LM (2005) Exposure to chronic stress downregulates corticosterone responses to acute stressors. Am J Physiol Regul Integr Comp Physiol 288: R1628–R1636. 15886358 10.1152/ajpregu.00484.2004

[cow049C36] Sapolsky RM, Romero LM, Munck AU (2000) How do glucocorticoids influence stress responses? Integrating permissive, suppressive, stimulatory, and preparative actions. Endocr Rev 21: 55–89. 10696570 10.1210/edrv.21.1.0389

[cow049C37] Siebert U, Pozniak B, Hansen KA, Nordstrom G, Teilmann J, van Elk N, Vossen A, Dietz R (2011) Investigations of thyroid and stress hormones in free-ranging and captive harbor porpoises (*Phocoena phocoena*): a pilot study. Aquat Mamm 37: 443–453.

[cow049C38] Small MF, Rosales R, Baccus JT, Weckerly FW, Phalen DN, Roberson JA (2004) A comparison of effects of radiotransmitter attachment techniques on captive white-winged doves. Wildlife Soc B 32: 627–637.

[cow049C39] Stauss H (2003) Heart rate variability. Am J Physiol Regul Integr Comp Physiol 285: R927–R931. 14557228 10.1152/ajpregu.00452.2003

[cow049C40] Terio K, Marker L, Munson L (2004) Evidence for chronic stress in captive but not free-ranging cheetahs (*Acinonyx jubatus*) based on adrenal morphology and function. J Wildl Dis 40: 259–266. 15362825 10.7589/0090-3558-40.2.259

[cow049C41] von Borell E, Langbein J, Després G, Hansen S, Leterrier C, Marchant-Forde J, Marchant-Forde R, Minero M, Mohr E, Prunier A et al (2007) Heart rate variability as a measure of autonomic regulation of cardiac activity for assessing stress and welfare in farm animals – a review. Physiol Behav 92: 293–316. 17320122 10.1016/j.physbeh.2007.01.007

[cow049C42] Wingfield J, Smith J, Farner D (1982) Endocrine responses of white-crowned sparrows to environmental stress. Condor 84: 399–409.

[cow049C43] Wingfield J, Vleck C, Moore M (1992) Seasonal changes of the adrenocortical response to stress in birds of the Sonoran desert. J Exp Zool 264: 419–428. 1460439 10.1002/jez.1402640407

[cow049C44] Zachariasen RD, Newcomer WS (1974) Phenylethanolamine-*N*-methyl transferase activity in the avian adrenal following immobilization or adrenocorticotropin. Gen Comp Endocrinol 23: 193–198. 4366215 10.1016/0016-6480(74)90128-2

